# Cross-sectional analysis of 152 hyperthyroid cats presented for radioactive iodine therapy in South Korea

**DOI:** 10.3389/fvets.2026.1906840

**Published:** 2026-10-01

**Authors:** Dasom Son, Yeon Chae, Taesik Yun, Hakhyun Kim, Jae-Cheong Lim, Byeong-Teck Kang

**Affiliations:** 1 Laboratory of Veterinary Internal Medicine, College of Veterinary Medicine, Chungbuk National University, Cheongju, Chungbuk, Republic of Korea; 2 Radioisotope Research Division, Korea Atomic Energy Research Institute, Daejeon, Republic of Korea

**Keywords:** Asia, epidemiology, feline hyperthyroidism, radioiodine therapy, referral patterns

## Abstract

**Introduction:**

Feline hyperthyroidism is one of the most common endocrinopathies in cats; however, its epidemiological characteristics remain poorly understood in South Korea. Although radioactive iodine therapy (RAIT) is a definitive treatment, studies focusing specifically on cats with hyperthyroidism undergoing RAIT are limited.

**Methods:**

Medical records of 152 cats with hyperthyroidism presented for RAIT were retrospectively reviewed to describe their epidemiological and clinical features.

**Results:**

The median age at diagnosis was 12 years, and 30.3% of the cats were diagnosed incidentally. RAIT was usually performed soon after diagnosis, with a median disease duration of 5 months; only 4.6% of cats had a disease duration >2 years. Unilateral thyroid disease was the predominant pattern (50.7%), and severe hyperthyroidism (total thyroxine ≥19.4 μg/dl) was observed in 10.5% of cats. Only 40.1% of cats underwent RAIT following the veterinarian's recommendation, whereas 59.9% of owners sought RAIT independently. Human-licensed or generic methimazole was used more frequently than veterinary-licensed methimazole (84.0 vs. 18.1%). Pruritus occurred more frequently with human-licensed or generic methimazole than with veterinary-licensed methimazole (*p* = 0.047). All cats were housed indoors. The regional distribution of cases differed from the reported national distribution of owned cats (*p* = 0.016). No cats originated from Jeju-do, which differed significantly from the mainland regions (*p* = 0.0004). Longer disease duration was associated with more advanced disease, including higher thyroid hormone levels and scintigraphic activity, increased bilateral involvement, and a higher rate of refractory response to antithyroid drugs (all *p* ≤ 0.001).

**Conclusions:**

This is the first study to characterize cats with hyperthyroidism that were presented for RAIT in South Korea. Key findings included an owner-driven pattern of RAIT selection, early use of definitive therapy despite a relatively late diagnosis, predominant use of non-veterinary methimazole formulations, and a higher frequency of pruritus as an adverse effect. These findings contribute to the epidemiological understanding of feline hyperthyroidism and may inform clinical decision-making in South Korea.

## 1 Introduction

Feline hyperthyroidism (FHT) is the most common endocrine disorder in older cats and is characterized by the autonomous overproduction of thyroid hormones, usually due to benign nodular hyperplasia or adenoma of the thyroid gland (1–3).

Over the past decades, numerous epidemiological studies have examined the prevalence and risk factors for FHT in different regions (4–6). These investigations have identified advanced age, non-purebred status, and an indoor lifestyle as consistent host-related risk factors. Dietary patterns and environmental exposure have also been implicated. Certain canned foods, particularly those with fish or liver flavors, and imbalances in dietary iodine intake have been associated with an increased risk of FHT (7–9). In addition, chronic exposure to environmental thyroid-disrupting chemicals, including polybrominated diphenyl ethers, polychlorinated biphenyls, and other persistent organic pollutants, has been proposed as a contributor to the development of nodular thyroid disease in susceptible cats (10–14). Collectively, these findings suggest that lifestyle, nutrition, and environmental contamination have interacted over many years to drive the current epidemiological patterns of FHT (3).

Several treatment options are available for FHT, including antithyroid drugs, iodine-restricted diets, surgical thyroidectomy, and radioactive iodine therapy (RAIT) (15–17). Among these, RAIT is widely regarded as the treatment of choice for most hyperthyroid cats because a single administration achieves a high rate of durable remission with a relatively low risk of serious adverse effects (18–20). Several clinical studies on FHT have been conducted in cohorts of cats treated with RAIT at referral centers, although most have been reported in North America or Europe (18–23). However, these studies have primarily focused on treatment protocols, predictors of outcomes, and renal effects, rather than a comprehensive epidemiological description of the underlying hyperthyroid population.

Importantly, the reported prevalence, signalment, and clinical phenotypes of FHT vary between countries and regions within the same country (24–26). Studies from New Zealand, North America, Europe, and South Africa have described differences in the proportion of affected purebred cats, typical age at diagnosis, sex distribution, and feeding practices (5, 6, 8, 9, 27, 28). These geographic variations likely reflect differences in cat-keeping practices, dietary preferences, environmental chemical profiles, access to veterinary care, study designs, and study populations. Consequently, epidemiological data from one region cannot be generalized to cats living under different conditions.

In South Korea, the population of companion cats has grown rapidly in recent years, with a high proportion of strictly indoor cats and distinctive patterns of urban housing, nutrition, and environmental exposure compared to those in Western countries (29). RAIT has become increasingly available, and its safety and efficacy in South Korea have been documented (30). However, no large-scale study has provided a detailed description of cats with hyperthyroidism undergoing RAIT in South Korea. Epidemiological data on FHT from Asia are also limited and largely confined to studies from Hong Kong and Japan, which were conducted in different clinical settings and therefore may not reflect the characteristics of cats presenting with RAIT in South Korea (24, 31).

Given the geographic variation in risk factors and disease expression, it remains uncertain whether hyperthyroid cats in South Korea exhibit epidemiological patterns similar to those reported in other regions of the world. In particular, the clinical presentation of cats undergoing evaluation for RAIT in South Korea, including the factors influencing treatment selection and patterns of previous antithyroid drug (ATD) use, has not been investigated in a large cohort. Therefore, this study aimed to describe the epidemiological and clinical features of cats with hyperthyroidism presented for RAIT in South Korea.

## 2 Materials and methods

### 2.1 Animals

A retrospective cross-sectional review was conducted of the medical records of cats presented for RAIT at Chungbuk National University Veterinary Teaching Hospital (Cheongju, South Korea) between July 2018 and September 2025. Data were collected regarding signalment (age, sex, neuter status, and breed), history, clinical signs, physical examination findings, total thyroxine (tT4) levels, and scintigraphic findings. Body condition score was evaluated for each cat on a validated 9-point scale, ranging from 1 (emaciated) to 9 (severely obese), with a score of 5 considered ideal (32). To compare the purebred and mixed-breed groups, Korean Shorthair, American Shorthair, British Shorthair, and cats recorded as mixed breeds were analyzed as the mixed-breed group.

The inclusion criteria were a confirmed diagnosis of hyperthyroidism based on compatible clinical signs, elevated serum tT4 concentration with low thyroid-stimulating hormone (TSH) levels, and increased thyroid-to-salivary ratio (TSR) and thyroid-to-background ratio (TBR) on thyroid scintigraphy. Cats with incomplete medical records or euthyroid status at the time of presentation were excluded. Methimazole was discontinued at least 1 week before the examination to allow assessment of thyroid status by serum thyroid hormone testing and scintigraphy.

### 2.2 Housing environment and geographic distribution

Housing status was categorized as strictly indoor, outdoor with regular access, allowed outdoors in a garden under supervision, or allowed outdoors on a leash only (33). Geographic locations were classified according to administrative divisions, including special cities, metropolitan cities, and provinces (29). One cat that had immigrated from the United States just before presentation was classified as originating overseas.

### 2.3 Clinical findings

A standardized set of 18 clinical signs associated with FHT (weight loss, polyphagia, increased activity/restlessness, polydipsia/polyuria, vomiting, diarrhea, increased fecal volume, anorexia, polypnea, muscle weakness, muscle tremor, increased nail growth, dyspnea, alopecia, tachycardia, cardiac murmur, congestive heart failure, and ventroflexion of the neck) (34) was evaluated in all cats based on owner history and physical examination. Tachycardia was defined as a heart rate of >220 beats/min. Clinical severity was scored from 1 to 3 according to the number of clinical findings present (score 1, 1–6 signs; score 2, 7–12 signs; score 3, 13–18 signs) (18, 30).

### 2.4 Treatment history

Disease duration was defined as the time interval between diagnosis and presentation for RAIT. Disease duration was categorized using a modified version of a previously reported scheme (35). Group 1 included cats diagnosed within 1 year before presentation for RAIT, and group 2 comprised those diagnosed 1–2 years before presentation. Because of the small number of cats with a longer disease duration, groups 3–5 (2–3, 3–4, and >4 years) were combined into a single category (group 3, >2 years).

Hyperthyroidism was considered incidentally diagnosed when increased serum T4 levels were detected during evaluation for reasons unrelated to the clinical signs of hyperthyroidism (e.g., vaccination or routine health examination).

Treatments attempted before RAIT, reasons for choosing RAIT, and the route of presentation were recorded. For cats that were administered ATDs, the drugs used, treatment response, and adverse effects were reviewed. Methimazole was categorized as veterinary-licensed methimazole (Felimazole^®^ Dechra, USA) or human-licensed/generic methimazole. A refractory response was defined as an initial response to methimazole (normalization of serum T4), followed, over a period of weeks to months, by a gradual decline in thyroid control despite an increase in the daily methimazole dose (35). The route of presentation for RAIT was classified as veterinarian- or owner-driven.

### 2.5 Thyroid scintigraphy

Thyroid scintigraphy was performed to assess the pattern of thyroid involvement (unilateral, bilateral, or multifocal disease), location of hyperfunctional thyroid nodules (cervical, thoracic inlet, or intrathoracic), the presence of ectopic thyroid tissue, and thyroid function parameters (TSR and TBR) (35, 36). Multifocal disease was defined as the presence of three or more separate thyroid nodules or distinct areas of increased radionuclide uptake (35, 36). Ectopic thyroid tissue was defined as hyperfunctional thyroid tissue located outside its normal mid-ventral cervical location (35, 36). Images were obtained using a breast-specific gamma camera (Dilon 6,800 Gamma Camera; Dilon Technologies, USA) with a low-energy, high-resolution, 15-degree slant parallel-hole collimator (37). Following intravenous injection of 111–148 MBq of technetium pertechnetate (^99m^${\text{TcO}}_{4}^{-}$; New Korea Industrial, Korea), three views of the thyroid (dorsoventral, right, and left lateral recumbency) were obtained after an interval of 20–60 min. Each image was acquired for a total of 200,000 counts. The images were integrated and processed using dedicated software (Dilon 6,800).

The TSR was calculated by dividing the average thyroid count density from both thyroid lobes (total thyroid counts/total thyroid pixels) by the average salivary count density (total salivary counts/total salivary gland pixels) (38). Similarly, the TBR was calculated by dividing the average thyroid count density (total thyroid counts/total thyroid pixels) by the average background count density (total background counts/total background gland pixels) (39). Previously reported reference intervals were adopted (TSR, 0.5–1.5; TBR, 1.6–6.4) (36). The number and anatomical location of foci with increased ^99m^${\text{TcO}}_{4}^{-}$ uptake were recorded. For cases of unilateral disease or unifocal ectopic tissue, increased uptake was defined as a TSR >1.5, whereas TBR was used as a complementary quantitative parameter. For bilateral or multifocal disease, if any focus had a TSR >1.5, additional simultaneous foci were also classified as abnormal, even when the TSR was < 1.5, indicating inappropriate uptake due to the loss of normal feedback suppression.

### 2.6 Thyroid hormone

Serum tT4 concentration was evaluated on the day of admission for RAIT and measured using an enzyme immunoassay analyzer (Immulite 2,000; Siemens Healthcare Diagnostics, USA), which was validated for use in cats (30, 40, 41). When the analyzer reported a value above the instrument's upper reportable limit (>15 μg/dl), samples were submitted to a commercial laboratory (IDEXX Laboratories, Inc., USA) for quantitation. The severity of hyperthyroidism was categorized as mild (< 9.6 μg/dl), moderate (9.7–19.3 μg/dl), or severe (≥19.4 μg/dl), based on serum tT4 levels (23).

### 2.7 Statistical analysis

All statistical analyses were performed using GraphPad Prism software (version 10.0; GraphPad Software). Data were assessed for normality using the Kolmogorov–Smirnov test, and nonparametric tests were used because the assumption of a Gaussian distribution was not met. Continuous variables were analyzed descriptively and were presented as medians and interquartile ranges (IQR) and illustrated as box plots or bar graphs. Exact binomial tests were used to compare the observed proportions of males vs. females and purebred vs. mixed-breed cats, with an expected proportion of 0.5. The regional distribution of cases was compared with the reported national distribution of owned cats using a chi-square goodness-of-fit test with a Monte Carlo simulation (1,000,000 replicates, without continuity correction). Chi-square or Fisher's exact tests were used, as appropriate, to compare categorical variables between groups, including tumor pattern according to disease duration, methimazole formulation according to treatment response or adverse effects, and refractory response to ATDs according to disease duration and hyperthyroidism severity based on serum tT4 concentration. When an overall difference among three groups was significant, *post hoc* pairwise comparisons were performed using Fisher's exact or chi-square tests, as appropriate. The Kruskal–Wallis test was used to compare continuous variables (serum tT4 concentration, TSR, and TBR) among the disease duration groups, followed by Dunn's multiple comparison test for *post hoc* pairwise comparisons. In all statistical analyses, *p* < 0.05 was considered significant.

## 3 Results

### 3.1 Animals

A total of 152 cats met the inclusion criteria (Figure 1). The demographic characteristics of the cats are summarized in Table 1. The median age at diagnosis of FHT and presentation for RAIT was 12 years in both groups. Among 152 cats, 30 cats (19.7%) were diagnosed with FHT before 10 years of age, of which 25 (16.4%) were presented for RAIT before reaching 10 years of age. The cohort included 79 (52.0%) females and 73 (48.0%) males, with no significant difference compared to the expected equal proportions of females and males (*p* > 0.05). The most common breed was Korean Shorthair (*n* = 77, 50.7%), followed by Russian Blue (*n* = 19, 12.5%), Persian (*n* = 14, 9.2%), and Siamese (*n* = 10, 6.6%) cats. Overall, 66 (43.4%) cats were purebred and 86 (56.6%) were mixed-breed cats, with no significant differences from the expected proportion of 0.5 (*p* > 0.05).

**Figure 1:**
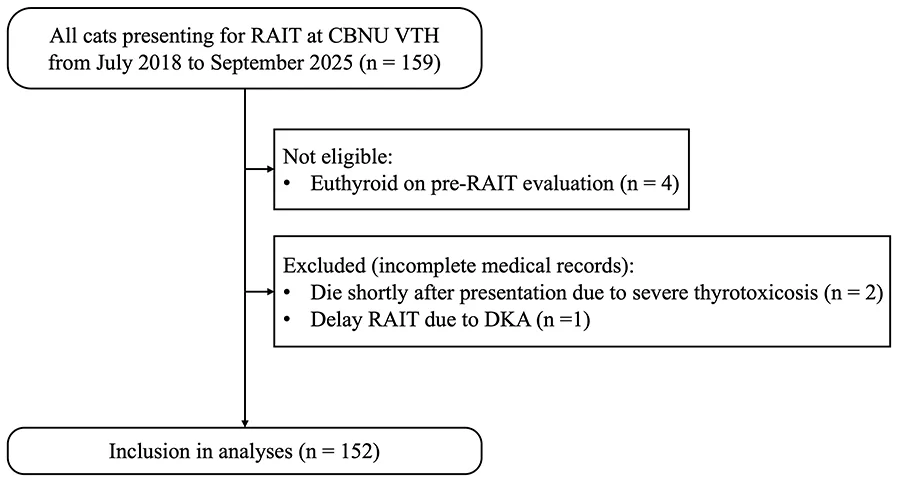
Flowchart of the study inclusion criteria. The medical records of 159 cats presented for radioactive iodine therapy (RAIT) at Chungbuk National University Veterinary Teaching Hospital (CBNU VTH) between July 2018 and September 2025 were reviewed. Four cats were excluded because they were euthyroid on the pre-RAIT evaluation, and three were excluded because of incomplete medical records. A total of 152 cats met the inclusion criteria and were included in the analysis. DKA, diabetic ketoacidosis.

**Table 1:** Demographic characteristics of 152 cats with hyperthyroidism presenting for RAIT.

Characteristic	Median (IQR) or n (%)
Age (years)	
At presentation for RAIT	12 (10–14)
At diagnosis of FHT	12 (10–13)
Sex	
Spayed female	76 (50.0)
Castrated male	70 (46.1)
Intact female	3 (2.0)
Intact male	3 (2.0)
Breed	
Korean shorthair	77 (50.7)
Russian blue	19 (12.5)
Persian	14 (9.2)
Siamese	10 (6.6)
Turkish angora	6 (3.9)
American shorthair	5 (3.3)
Norwegian forest cat	4 (2.6)
Scottish fold	4 (2.6)
Bengal	3 (2.0)
Neva masquerade	2 (1.3)
Ragdoll	2 (1.3)
British shorthair	2 (1.3)
Mixed breed	2 (1.3)
Scottish straight	1 (0.7)
Sphynx	1 (0.7)
Body condition and vital signs	
Body weight (kg)	4.3 (3.7–5.5)
Body condition score (/9)	5 (4–6)
Heart rate (beats/minute)	222 (192–224)
Blood pressure (mmHg)	150 (140–160)
Body temperature (°C)	38.6 (38.4–39.1)

Continuous variables (age, body condition, and vital signs) are presented as medians (interquartile range). Categorical variables (sex and breed) are presented as the number of cats (%). To compare purebred and mixed-breed cats, Korean Shorthair, American Shorthair, British Shorthair, and cats recorded as mixed breeds are classified as mixed-breed. RAIT, radioactive iodine therapy; FHT, feline hyperthyroidism.

### 3.2 Housing environment and geographic distribution

All 152 cats were strictly kept indoors. Of the 152 hyperthyroid cats, 151 were from South Korea and were compared with the regional distribution of the owned cat population (29) as follows: Seoul and Gyeonggi-do, followed by Busan, Gyeongsangnam-do, Daejeon, Incheon, Chungcheongbuk-do, Daegu, Jeollabuk-do, Gyeongsangbuk-do, Ulsan, Chungcheongnam-do, Jeollanam-do, Gwangju, Gangwon-do, Sejong, and Jeju-do (Table 2 and Figure 2). One additional cat (0.7%) originated overseas (the United States). The geographic distribution of the 151 cats differed significantly from the reported national distribution of owned cats (*p* = 0.016; Table 2) (29). In a comparison between Jeju-do and the mainland regions, no cats originated from Jeju-do (0/151), although 12.2 cats were expected based on the national distribution of owned cats (Fisher's exact test, *p* = 0.0004).

**Table 2:** Geographic distribution of hyperthyroid study cats compared with the national owned cat distribution.

Region in South Korea	National distribution of hyperthyroid cats			National distribution of owned cats		
	Rank	Population (*n*)	Percentage (%)	Rank	Population (*n*)	Percentage (%)
Seoul	1	46	30.3	2	13,434	23.6
Gyeonggi-do	2	41	27.0	1	16,774	29.4
Busan	3	11	7.2	7	2,032	3.6
Gyeongsangnam-do	4	8	5.3	8	1,903	3.3
Daejeon	5	7	4.6	5	2,786	4.9
Chungcheongbuk-do	6	6	3.9	11	1,603	2.8
Incheon	7	6	3.9	6	2,275	4
Daegu	8	5	3.3	4	2,793	4.9
Jeollabuk-do	9	5	3.3	12	1,125	2
Gyeongsangbuk-do	10	4	2.6	9	1,859	3.3
Chungcheongnam-do	11	3	2.0	10	1,807	3.2
Jeollanam-do	12	3	2.0	17	608	1.1
Ulsan	13	3	2.0	15	727	1.3
Gwangju	14	2	1.3	13	1,060	1.9
Gangwon-do	15	1	0.7	14	963	1.7
Sejong	16	0	0.0	16	633	1.1
Jeju-do	17	0	0.0	3	4,601	8.1

Values are presented as numbers and percentages. Of the 152 study cats, 151 originated from South Korea; one cat originating from the United States is not shown in the table. The percentages for the study cats are based on all 152 cats. Data on the national distribution of owned cats were obtained from the 2025 Korea Companion Animal Report (29). The regional distribution of the study cats differs significantly from the national distribution of owned cats (chi-square goodness-of-fit test with Monte Carlo simulation; 1,000,000 replicates without continuity correction; *p* = 0.016).

**Figure 2:**
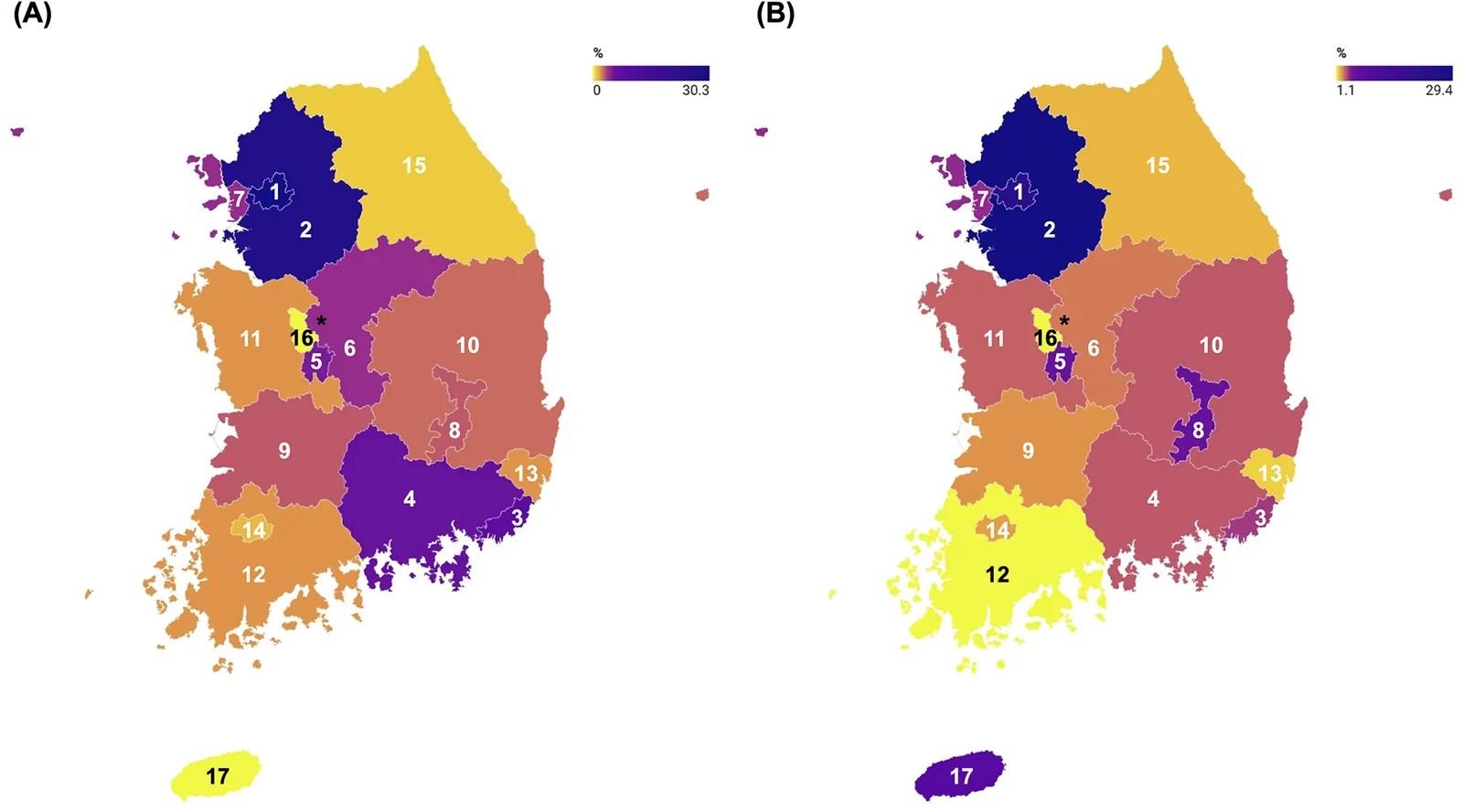
Geographic distribution of hyperthyroid study cats compared with the national owned cat distribution. **(A)** Proportion of the 152 cats in this study originating from each administrative region. **(B)** Proportion of owned cats in each region in the general population, based on the 2025 Korea Companion Animal Report (29). Colors represent the regional proportion of cats, with higher values shown toward the upper end of the scale in each panel. Numbers identify the administrative regions, ordered according to the proportion of study cats: 1, Seoul; 2, Gyeonggi-do; 3, Busan; 4, Gyeongsangnam-do; 5, Daejeon; 6, Chungcheongbuk-do; 7, Incheon; 8, Daegu; 9, Jeollabuk-do; 10, Gyeongsangbuk-do; 11, Chungcheongnam-do; 12, Jeollanam-do; 13, Ulsan; 14, Gwangju; 15, Gangwon-do; 16, Sejong; and 17, Jeju-do. The detailed population distribution for each region is provided in Table 2.

### 3.3 Clinical findings

Among the 152 cats, the median number of clinical findings was six (IQR, 4–8). Based on the clinical finding score, 87 (57.2%) cats were classified as score 1, 63 (41.4%) as score 2, and two (1.3%) as score 3. The most frequently reported clinical findings were weight loss (88.2%), vomiting (67.8%), increased activity (66.4%), tachycardia (59.9%), polyphagia (55.9%), and polydipsia (55.3%). Other common abnormalities included tachypnea (36.8%), diarrhea (34.2%), alopecia (31.6%), anorexia (28.9%), and muscle weakness (25.0%). Less common findings were dyspnea (17.1%), increased fecal volume (15.8%), nail overgrowth (7.9%), muscle tremor (7.2%), cardiac murmur (7.2%), and ventroflexion of the neck (2.0%); congestive heart failure was not documented in any cat at presentation.

### 3.4 Treatment history

The median disease duration was 5 months (IQR, 3–10 months). Group 1 comprised 128 (84.2%) cats, group 2 comprised 17 (11.2%), and group 3 comprised seven (4.6%).

Hyperthyroidism was incidentally diagnosed in 46 (30.3%) cats. Before presentation, 144 cats had received ATDs, two had been managed with an iodine-restricted diet (Prescription Diet y/d; Hill's Pet Nutrition, USA), and one had previously undergone RAIT at another hospital. In 87 (55.1%) cats, the decision to undergo RAIT was related to prior ATD therapy, most commonly due to adverse effects, difficulties in medication administration, and inadequate hyperthyroidism control (Table 3). Among the 144 cats that received antithyroid medication before RAIT, a refractory response to treatment was documented in 30.6% of the cats (Table 3). ATD administration differed significantly according to disease duration (Fisher's exact test, *p* = 0.0037; Table 4). All cats in groups 2 and 3 received ATDs (100%), whereas 120 of the 128 cats in group 1 received ATDs (93.8%; Fisher's exact test for group 1 vs. groups 2 and 3, *p* = 0.0289). The proportion of cats with a refractory response to ATDs also increased with longer disease duration (overall chi-squared test, *p* = 0.0008; Table 4). In pairwise comparisons, groups 2 and 3 had significantly higher refractory rates than group 1 (*p* = 0.0002 and 0.0177, respectively). Only 61 of the 152 cats (40.1%) underwent RAIT based on the recommendation of a veterinarian, whereas the remaining 91 (59.9%) underwent RAIT as an owner-driven choice.

**Table 3:** Reasons for choosing radioiodine therapy (RAIT) in 152 hyperthyroid cats.

Reason for choosing RAIT	*n* (%)
About antithyroid drug	87 (55.1)
Refractory response with medication	20 (12.7)
Adverse effect with medication	34 (21.5)
Medication administration issue	33 (20.9)
Desire for definitive cure	58 (36.7)
Others	2 (1.3)
Incomplete medical records	11 (7.0)

Values represent the number of cats (%). Reasons related to antithyroid drug treatment (refractory response, adverse effects, and difficulties with administration) are grouped under “About antithyroid drug.” The owners were allowed to report more than one reason for an individual cat (multiple responses were allowed). The reasons for RAIT were not documented in 11 cats (incomplete medical records). RAIT, radioactive iodine therapy.

**Table 4:** Refractory response to ATDs according to disease duration and serum thyroid hormone in 152 hyperthyroid cats.

Disease duration					
Outcome	All cats	Group 1	Group 2	Group 3	*P*–value
Number of cats (%)	152 (100.0)	128 (84.2)	17 (11.2)	7 (4.6)	–
Cats administered ATDs (%)	144 (94.7)	120 (93.8)	17^a^ (100.0)	7^a^ (100.0)	0.0037^∫^
Refractory response to ATDs (%)	44 (30.6)	32 (26.7)	9^b^ (52.9)	3^c^ (42.9)	0.0008^∫∫^
Serum total T4 concentration					
Outcome	All cats	Mild	Moderate	Severe	*p*-value
Number of cats (%)	152 (100.0)	42 (27.6)	94 (61.8)	16 (10.5)	–
Cats administered ATDs (%)	144 (94.7)	37 (88)	91 (96.8)	16 (100.0)	0.1219
Refractory response to ATDs (%)	44 (30.6)	4 (10.8)	31^d^ (34.1)	9^e^ (56.3)	0.0013^*^

Values represent the number of cats (%). The percentages for “Cats administered ATDs” are based on all cats in each group, whereas percentages for “Refractory response to ATDs” are based on only those cats that received ATDs. Group 1: diagnosed < 1 year before RAIT. Group 2: diagnosed 1–2 years before RAIT. Group 3: diagnosed >2 years before RAIT. Hyperthyroidism severity was classified according to serum tT4 concentration as mild (< 9.6 μg/dL), moderate (9.7–19.3 μg/dl), or severe (≥19.4 μg/dl).

^∫^*p*-value calculated using Fisher's exact test (overall comparison among groups).

^∫∫^*p* value calculated using the chi-square test (overall comparison among groups).

^*^p value calculated using Fisher's exact test (overall comparison among groups).

^a^Significantly different from group 1 (Fisher's exact test, *p* = 0.0289).

^b^Significantly different from group 1 (chi-square test, *p* = 0.0002).

^c^Significantly different from group 1 (chi-square test, *p* = 0.0177).

^d^Significantly different from the mild group (Fisher's exact test, *p* = 0.0082).

^e^Significantly different from the mild group (Fisher's exact test, *p* = 0.0010).

ATD, antithyroid drug; RAIT, radioactive iodine therapy.

Among the 144 cats that received ATDs before RAIT, 26 (18.1%) were treated with veterinary-licensed methimazole and 121 (84.0%) were treated with human-licensed or generic methimazole (Table 5). No significant difference was observed between veterinary-licensed and human-licensed/generic methimazole in terms of the number of cats showing refractory responses or adverse effects (*p* = 0.483 and 0.245, respectively). Pruritus (20.8%) was the most common adverse effect and occurred significantly more frequently in cats receiving human-licensed or generic methimazole than in those receiving veterinary-licensed methimazole (*p* = 0.047). Other adverse events were infrequent and showed no significant differences between the groups (*p* > 0.05).

**Table 5:** Treatment response and adverse effects according to methimazole formulation in 144 cats treated with ATDs.

Outcome	All cats administered ATDs	Veterinary-licensed methimazole	Human-licensed or generic methimazole	*p-*value
Number of cats (%)	144 (100.0)	26 (18.1)	121 (84.0)	
Treatment response				
Refractory response (%)	44 (30.6)	6 (23.1)	39 (32.2)	0.483
Adverse effects				
**Adverse effects (%)**	42 (29.2)	5 (19.2)	38 (31.4)	0.245
Pruritus	30 (20.8)	1 (3.8)	25 (20.7)	0.047^*^
Alopecia	8 (5.6)	1 (3.8)	7 (5.8)	>0.999
Gastrointestinal upset	8 (5.6)	1 (3.8)	7 (5.8)	>0.999
Anorexia	4 (2.8)	1 (3.8)	3 (2.5)	0.680
Elevated liver enzymes	6 (4.2)	1 (3.8)	5 (4.1)	>0.999
Polydipsia/polyuria	1 (0.7)	0 (0.0)	1 (0.8)	>0.999
Hematologic abnormalities	4 (2.8)	0 (0.0)	4 (3.3)	>0.999
Others	3 (2.1)	0 (0.0)	3 (2.5)	>0.999

Values represent the number of cats (%). “All cats administered ATD” includes all 144 cats that received methimazole. Some cats changed their formulations during treatment; therefore, the same cat may have been counted in both formulation columns. Cats experiencing more than one adverse effect are included in each category. The *p*-values compare veterinary-licensed with human-licensed or generic methimazole (Fisher's exact test, ^*^p < 0.05). ATD, antithyroid drug; RAIT, radioactive iodine therapy.

### 3.5 Thyroid scintigraphy

Among the 152 cats, representative scintigraphic uptake patterns are presented in Figure 3. Unilateral thyroid disease was the predominant tumor pattern, identified in 77 (50.7%), whereas bilateral and multifocal diseases were observed in 66 (43.4%) and 9 (5.9%), respectively (Table 6). The percentage of unilateral disease declined, and the percentage of bilateral disease increased with increasing disease duration (*p* < 0.0001). Of the 238 hyperfunctional thyroid nodules identified on scintigraphy, 233 were located in the cervical region, 3 at the thoracic inlet, and 2 were intrathoracic.

**Figure 3:**
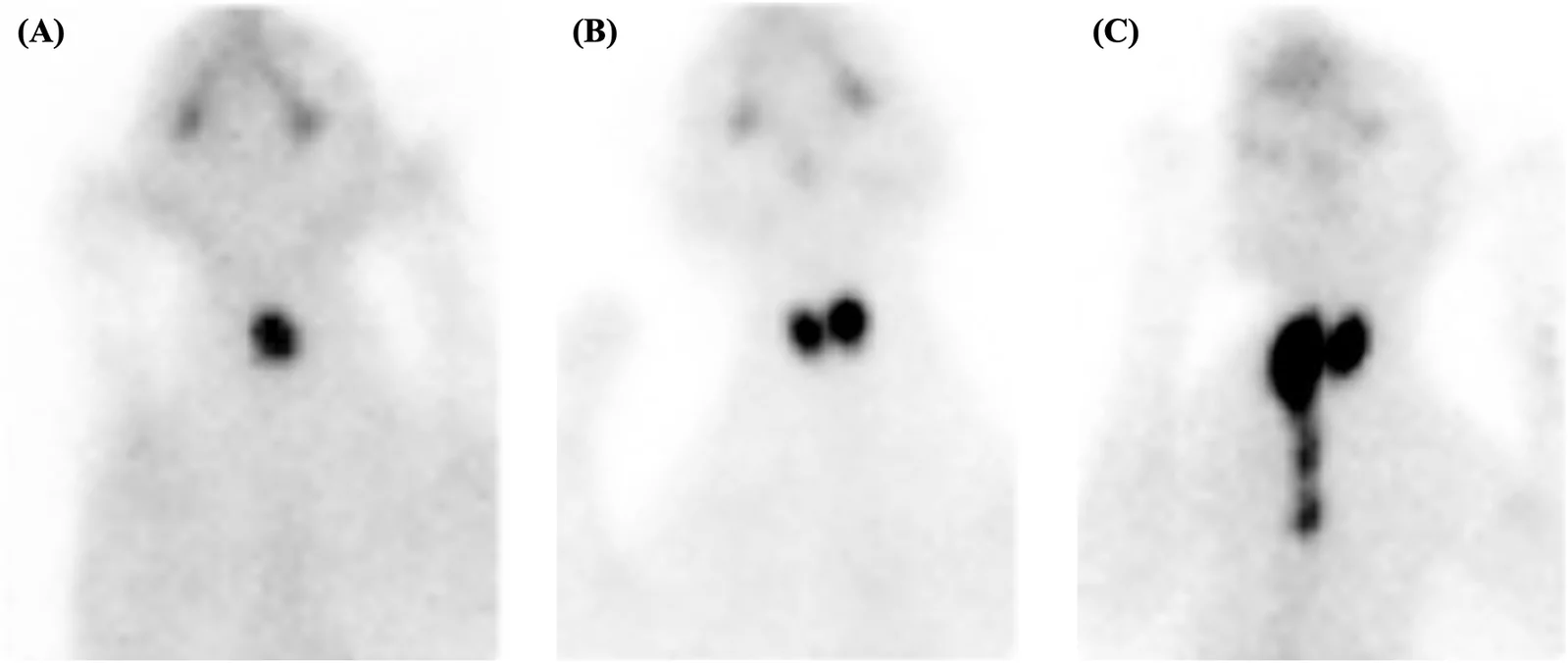
Representative thyroid scintigraphic patterns in cats with hyperthyroidism. **(A)** Unilateral disease involving a single thyroid lobe. **(B)** Bilateral disease involving both thyroid lobes. **(C)** Multifocal disease with three or more separate thyroid nodules or distinct areas of increased radionuclide uptake.

**Table 6:** Thyroid tumor pattern according to disease duration in 152 hyperthyroid cats.

Disease duration	All cats	Group 1	Group 2	Group 3	*p*-value
Number of cats (%)	152 (100.0)	128 (84.2)	17 (11.2)	7 (4.6)	–
Tumor pattern					
Unilateral	77 (50.7)	68 (53.1)	8 (47.1)	1^ab^ (14.3)	< 0.0001^*^
Bilateral	66 (43.4)	54 (42.2)	7 (41.2)	5^ab^ (71.4)	< 0.0001^*^
Multifocal	9 (5.9)	6 (4.7)	2 (11.8)	1^c^ (14.3)	0.090

Values represent the number of cats (%). Group 1: diagnosed < 1 year before RAIT. Group 2: diagnosis 1–2 years before RAIT. Group 3: diagnosis >2 years before RAIT. ^*^p-values are calculated using the chi-square test (overall comparison among groups for each tumor pattern).

^a^Significantly different from group 1 (chi-square test, *p* < 0.0001).

^b^Significantly different from group 2 (chi-square test, *p* < 0.0001).

^c^Significantly different from group 1 (chi-square test, *p* = 0.030).

RAIT, radioactive iodine therapy.

TSR and TBR tended to increase significantly with longer disease duration (*p* = 0.001 and *p* < 0.0001, respectively). The median TSR was significantly higher in groups 2 and 3 than in group 1 (*p* = 0.018 and *p* = 0.020, respectively) (Figure 4A). The median TBR was also significantly higher in groups 2 and 3 than in group 1 (*p* = 0.001 and *p* = 0.039, respectively) (Figure 4B).

**Figure 4:**
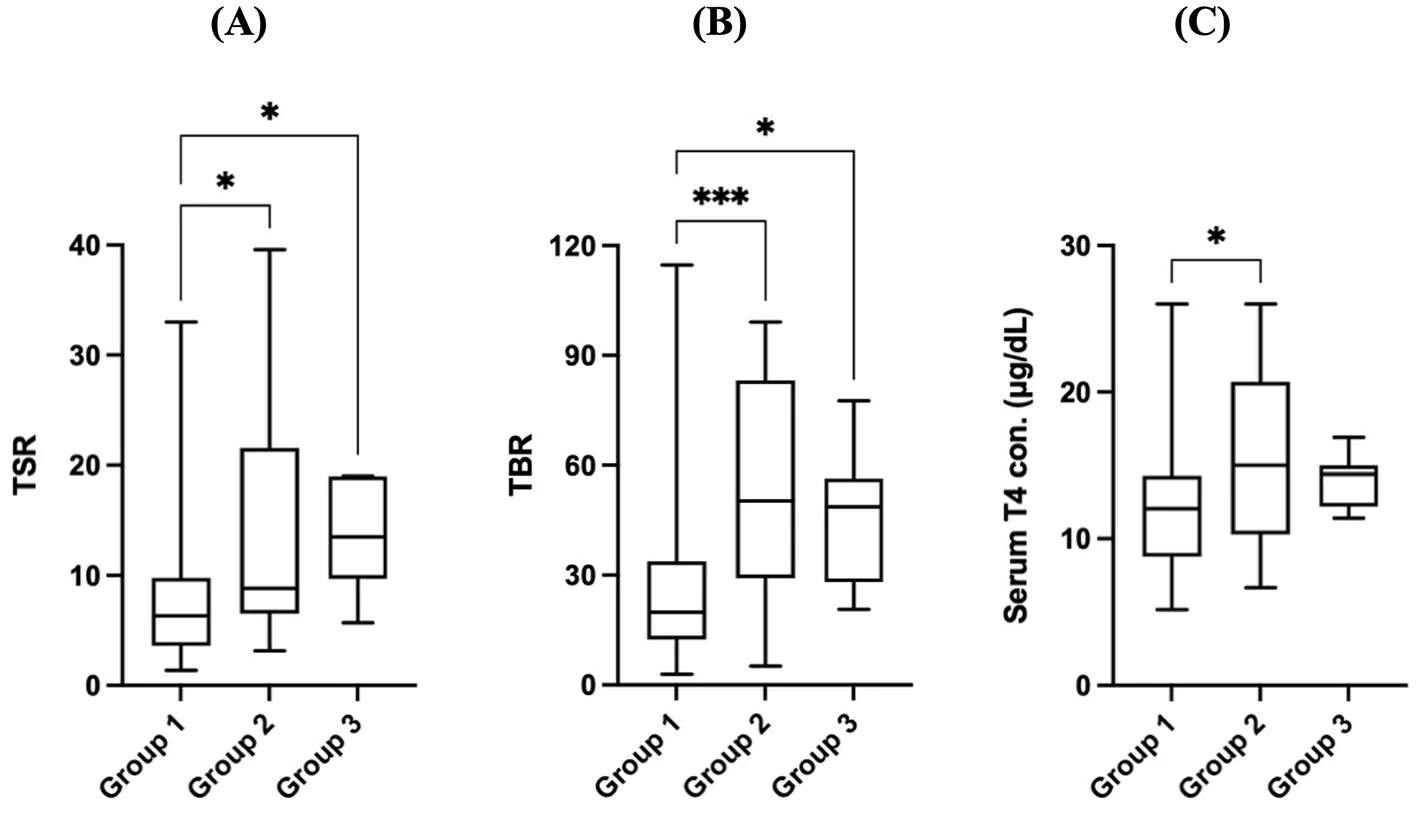
Thyroid scintigraphy parameters and serum total thyroxine (tT4) levels according to disease duration group. **(A)** Thyroid-to-salivary ratio (TSR). **(B)** Thyroid-to-background ratio (TBR). **(C)** Serum tT4 concentration. Group 1, diagnosis <1 year before radioiodine therapy (RAIT). Group 2, diagnosis 1–2 years before RAIT. Group 3, diagnosis >2 years before RAIT. Boxes represent the interquartile range, horizontal lines indicate medians, and whiskers indicate the range. Overall, all three variables differ significantly among the groups (Kruskal–Wallis test; tT4, *p* = 0.001; TSR, *p* = 0.001; TBR, *p* < 0.0001). On *post hoc* analysis (Dunn's test), tT4, TSR, and TBR are higher in cats with longer disease duration, with significant pairwise differences, as indicated by asterisks (*p < 0.05, ***p < 0.001).

### 3.6 Thyroid hormone

The median serum tT4 concentration for all 152 cats was 12.4 μg/dl (IQR, 9.4–14.8). Based on serum tT4 concentrations, 42 (27.6%), 94 (61.8%), and 16 (10.5%) cats had mild, moderate, and severe hyperthyroidism, respectively. Serum tT4 concentrations increased significantly with longer disease duration (*p* = 0.001) and were significantly higher in group 2 than in group 1 (*p* = 0.023) (Figure 4C). However, serum tT4 concentrations did not differ significantly between groups 2 and 3 (*p* > 0.05). The proportion of cats with a refractory response increased with increasing hyperthyroidism severity based on serum total T4 concentration (overall Fisher's exact test, *p* = 0.0013; Table 4). In pairwise comparisons, the moderate and severe groups had significantly higher refractory rates than the mild group (*p* = 0.0082 and 0.0010, respectively).

## 4 Discussion

To the best of our knowledge, this is the first study to describe the epidemiological and clinical features of hyperthyroidism in cats in South Korea. Most of the epidemiological characteristics were similar to those reported in other countries. However, three clinical features were distinct in the present study. First, many cats underwent RAIT after their owners sought treatment, rather than through direct recommendations or referrals from primary care veterinarians. Second, human-licensed methimazole was used much more frequently than veterinary-licensed formulations. Third, although relatively few cats were diagnosed before 10 years of age, RAIT was usually administered soon after diagnosis rather than after prolonged medical management. Although data on FHT in Asian countries remain limited, this study appears to be the first to provide a detailed description of cats with hyperthyroidism treated with RAIT in Asia (24, 31).

The cohort included slightly more female than male cats; however, this difference was not significant. This is consistent with the results of most previous studies (4, 5, 24), although some authors have suggested a female predisposition (6, 8). Similar to toxic human nodular goiters, this may reflect the effects of sex hormones (42). Another possible explanation is sex-related differences in the metabolism of environmental toxicants, such as bisphenol A, which have been proposed as risk factors for FHT (43, 44). Differences in neutering age and rates between countries may also have influenced these findings (45).

As in previous studies, no significant difference between purebred and mixed-breed cats was observed (3, 4, 26). However, some studies have reported a lower risk of hyperthyroidism in purebred, short-haired cats, or specific breeds such as Siamese, Burmese, and Persian (6, 46). One hypothesis is that long-haired cats are more exposed to endocrine-disrupting chemicals because a larger haircoat surface allows more contaminants to accumulate and be ingested during grooming (46). In color-point breeds, reduced tyrosinase activity and melanin synthesis have been proposed to divert more tyrosine toward thyroid hormone production, which might modify susceptibility (46, 47). Korean Shorthair, Persian, and Siamese were among the most frequently represented breeds, and this pattern reflected the reported breed distribution of owned cats in South Korea (Korean Shorthair 44.7%, Russian Blue 12.8%, Persian 9.6%, Siamese 5.9%, and Turkish Angora 5.7%) (29). Thus, the breed distribution may reflect national ownership patterns rather than breed-specific differences in risk.

In this study, 19.7% of cats were diagnosed with hyperthyroidism before 10 years of age, which is lower than the 23.5%−34.9% reported in recent European cohorts from Ireland, the United Kingdom, and the Netherlands (23, 48, 49). Earlier reports described early onset disease as uncommon, with approximately 5% of cats being diagnosed before 10 years of age (50). Earlier and milder cases of hyperthyroidism are now diagnosed more often, probably because routine health-screening panels commonly include serum tT4, and TSH is measured more frequently (20, 51). However, fewer cats in this study were diagnosed with hyperthyroidism before 10 years of age, which may reflect the infrequent use of thyroid panels in routine health checks and limited perception of their importance among veterinarians and cat owners.

Hyperthyroidism was diagnosed incidentally in 30.3% of cats, compared with 2.3% in Belgium and 24.3% in the United Kingdom (23, 49). This relatively high proportion of incidental diagnoses may reflect the under-recognition of early clinical signs in older cats. Many owners and veterinarians may mistake the typical signs of FHT, such as weight loss, increased appetite, and restlessness, for normal aging-related changes (3, 16). This finding may also be consistent with the relatively low proportion of cats diagnosed before 10 years of age.

All cats included in this study were kept strictly indoors. Indoor living has been associated with a higher risk of FHT because house dust ingested during grooming increases exposure to endocrine-disrupting chemicals in indoor cats (14, 25). Environmental studies in Korea have reported relatively high concentrations of phthalate esters and brominated flame retardants in household dust, with levels in Asian homes (including Korea) often exceeding those reported in Europe and North America (52). In addition, higher body burdens of persistent organic pollutants have been associated with altered thyroid hormone concentrations in Korean women (53). Together, these findings raise the possibility that the incidence of FHT in strictly indoor Korean cats may be higher than that in some Western countries. In one household, both resident indoor cats developed hyperthyroidism approximately 1 year after wallpaper replacement. This anecdotal observation suggests a potential role for indoor environmental exposure. However, this study design does not allow estimation of the prevalence of FHT; therefore, direct comparisons between Korea and other regions are not possible.

Previous studies have suggested that travel distance and access to a radioiodine center are important factors when owners choose RAIT (33, 48). In South Korea, Jeju-do is separated from the mainland and is the region with the greatest travel distance (approximately 400 km) from the study institution in Cheongju, Chungcheongbuk-do. Therefore, cats living in Jeju-do must travel by airplane or ferry to receive radioiodine treatment. Owing to radiation safety guidelines after RAIT, the need to rely on public transport rather than private cars is a substantial practical barrier for owners from Jeju-do. Medication-related problems were a common reason for choosing RAIT in this study, and similar patterns have been reported in other studies (48). Only 40.1% of cats presented for RAIT based on veterinary recommendations, compared with 60% in the United Kingdom, 64% in Germany, and 82% in Belgium (33, 48, 49). This pattern may indicate that many owners in South Korea seek information regarding RAIT independently, although RAIT may not be consistently presented as a treatment option in primary care practice.

Although only two cats had a clinical finding score of 3, this should not be interpreted as indicating that cats with severe biochemical hyperthyroidism generally had mild clinical manifestations. All 16 cats with severe hyperthyroidism exhibited weight loss, and potentially more clinically significant findings, such as anorexia, polypnea, and cervical ventroflexion, were also proportionally more frequent in this group (Supplementary material 1). The proportion of cats with refractory responses to ATDs increased with increasing biochemical severity and was highest in the severe group. These findings suggest that biochemical severity based on serum tT4 concentration captures clinically relevant aspects of FHT that may not be fully reflected by a number-based clinical finding score. This interpretation is also consistent with previously proposed RAIT scoring approaches that incorporate serum tT4 concentration together with clinical assessment (54, 55). Existing clinical scoring systems have limitations in adequately capturing the true severity of FHT (54–56). Previous scoring approaches graded the overall severity of clinical signs from mild to severe without specifying standardized criteria for each severity grade (54, 55). Although a modified Karnofsky score has also been used to assess illness severity and quality of life in hyperthyroid cats, this index evaluates general health status and quality of life rather than the severity of individual hyperthyroidism-associated clinical findings (56). In the present study, the clinical finding score was based on the number of concurrent findings to enable quantitative comparison among cats (34). However, this approach did not account for the severity of individual findings. Therefore, the apparent discrepancy between the 16 cats with severe biochemical hyperthyroidism and the two cats with the maximum clinical finding score may partly reflect the limitations of a number-based scoring approach. Future studies may benefit from a standardized scoring system that incorporates the severity of individual clinical findings.

In South Korea, 84.0% of cats received human-licensed or generic methimazole before RAIT, whereas a previous survey from the United States and the United Kingdom reported that only 18.9% of cats with hyperthyroidism were treated with non-veterinary-licensed formulations (16). In the present study, pruritus was the most frequent adverse effect and occurred significantly more frequently in cats that received human-licensed or generic formulations than in those that received veterinary-licensed products. Previous studies have reported dermatological reactions in approximately 4% of methimazole-treated cats, whereas dermatological adverse effects were documented in 26.4% of cats in this study (20.8% with pruritus and 5.6% with alopecia) (17). One possible explanation is the low use of veterinary-licensed products, because differences in excipients may make pruritus less likely with veterinary-licensed formulations. For example, excipient-related cutaneous reactions, including focal pyotraumatic dermatitis and epidermal desquamation, have been described for levothyroxine in dogs, where inactive ingredients (magnesium stearate and polyvinylpyrrolidone), rather than the active hormone, are suspected to be the triggers (57). This pattern suggests that differences in tablet formulations, including excipient composition and the presence of a coating, may influence susceptibility to cutaneous reactions.

Only 4.6% of cats had a disease duration >2 years, which is lower than the 7.4 and 8.2% reported in studies from the United States and Germany, respectively, and similar to the 4.1% reported in Belgium (33, 35, 49). This may be partly explained by the fact that relatively few cats are diagnosed at an early stage in South Korea, as discussed above. This may also suggest a tendency to proceed to RAIT relatively soon after diagnosis rather than maintaining ATD therapy for several years.

In this study, unilateral thyroid disease was common, which is consistent with a relatively early referral for RAIT, and contrasts with many previous studies in which bilateral disease was more frequent (19, 36). In addition, severe hyperthyroidism was less frequent than that in a large United Kingdom RAIT cohort, with 10.5% of cats in the severe group in this study compared with 22.1% in that cohort (23). Although comparable data are not available from previous studies, only two cats (1.3%) were classified with a clinical sign score of 3, indicating that few cats exhibited a large number of clinical signs at the time of RAIT. Overall, these findings suggest that many cats were treated with RAIT at a relatively early stage of nodular thyroid disease before bilateral or multifocal involvement and marked biochemical severity had fully developed.

Longer disease duration was associated with higher serum tT4, TSR, and TBR, supporting the view that FHT is a progressive disease (1, 2). Simultaneously, unilateral thyroid disease became less common as disease duration increased, consistent with the progression to bilateral or multifocal involvement (36, 58). Refractory responses to methimazole were also more frequent in cats with a longer disease duration, indicating that medical control becomes less reliable as thyroid disease progresses. These patterns are consistent with previous evidence showing that chronic ATD therapy does not arrest the growth of hyperfunctional thyroid tissue (35, 59, 60). Over months to years, thyroid nodules tend to enlarge and develop additional autonomous foci, and some cats eventually lose medical control and require definitive treatment, such as thyroidectomy or RAIT (15, 18).

This study has some limitations. The retrospective single-center design may introduce selection and information bias and limit the generalizability of these findings to all hyperthyroid cats in South Korea. In addition, the reasons for choosing RAIT were abstracted from existing records rather than obtained through structured questionnaires; therefore, they may not fully capture the range and nuances of owners' motivations. Nevertheless, the impact of the single-center design should be considered in the context of the clinical landscape in South Korea. At the time of this report, RAIT was available at only two facilities nationwide, in Seoul and Cheongju, with most cases managed at the center in Cheongju. Accordingly, this cohort may reflect the national referral patterns for cats undergoing RAIT in South Korea.

## 5 Conclusions

This study provides the first detailed characterization of hyperthyroid cats presented for RAIT in South Korea and identifies several distinctive patterns compared with previously reported Western RAIT populations. Although the diagnosis was often made relatively late, RAIT was frequently undertaken early in the course of the disease, when involvement remained largely unilateral and most cats had not yet developed advanced clinical signs. A relatively small proportion of cases proceeded to RAIT based on veterinary recommendations, suggesting opportunities in primary care practice for routine thyroid screening in older cats and earlier discussions of definitive treatment options. In addition, the predominant use of non-veterinary methimazole formulations and the high frequency of dermatological adverse effects highlight the potential value of increasing the use of veterinary-licensed products.

## Data Availability

The raw data supporting the conclusions of this article will be made available by the authors, without undue reservation.
